# Cerebellar tDCS Does Not Improve Learning in a Complex Whole Body Dynamic Balance Task in Young Healthy Subjects

**DOI:** 10.1371/journal.pone.0163598

**Published:** 2016-09-26

**Authors:** Katharina Marie Steiner, Anne Enders, Wiebke Thier, Giorgi Batsikadze, Nicolas Ludolph, Winfried Ilg, Dagmar Timmann

**Affiliations:** 1 Department of Neurology, Essen University Hospital, University of Duisburg-Essen, Essen, Germany; 2 Cognitive Neurology, Section Computational Sensomotorics, Hertie Institute for Clinical Brain Research and Center for Integrative Neuroscience, Eberhard Karls University, Tübingen, Germany; University of Ottawa, CANADA

## Abstract

Transcranial direct current stimulation (tDCS) of the cerebellum is of increasing interest as a non-invasive technique to modulate motor performance and learning in health and disease. Previous studies have shown that cerebellar tDCS facilitates reach adaptation and associative motor learning in healthy subjects. In the present study it was tested whether cerebellar tDCS improves learning of a complex whole body motor skill. Because this task involves learning of posture and balance likely including learning of a new motor sequence and cognitive strategies, cerebellar tDCS was applied over midline cerebellar structures and the posterolateral cerebellar hemispheres. 30 young and healthy subjects performed two days of balance training on a Lafayette Instrument 16030 stability platform®. Participants received either anodal, cathodal or sham cerebellar tDCS during training on day 1. The cerebellar electrode (7 cm width by 5 cm height) was centered 2 cm below the inion. Mean platform angle deviation and mean balance time were assessed. All subjects showed significant effects of learning. Learning rate was not different between the three modes of stimulation neither on day 1 nor on day 2. Cerebellar tDCS did not facilitate learning of a complex whole body dynamic balance task in young and healthy subjects. tDCS effects, however, may have been missed because of the small group size. Furthermore, it cannot be excluded that young and healthy subjects learned and performed already at a near optimal level with little room for further improvement. Future work has to evaluate potential benefits of cerebellar tDCS in elderly subjects and subjects with cerebellar deficits, whose motor control and motor learning network is not optimally tuned.

## Introduction

Postural and balance functions play a crucial role in everyday activities such as standing and walking, especially on uneven ground. Adaptation to altered support conditions requires a complex coordination of trunk and multi-joint leg and arm movements. The cerebellum is known to play an important role in postural and balance control. Cerebellar disease results in ataxia of stance and gait, and disorders to adapt postural responses to unexpected perturbations [1–3]. Finding a tool to selectively enhance cerebellar functions in order to compensate for the poor postural and balance control would implicate great progress in the rehabilitation of cerebellar disease.

Transcranial direct current stimulation of the cerebellum is of increasing interest as a non-invasive technique to modulate motor performance and learning in health and disease [4]. Previous studies have shown that cerebellar tDCS has a beneficial effect on motor learning in several paradigms. Most studies investigated the effects of cerebellar tDCS on adaptation tasks. Anodal tDCS facilitated reach adaptation [5] and locomotor adaptation in young and healthy subjects [6]. Subjects receiving anodal cerebellar tDCS showed faster locomotor adaptation whereas cathodal tDCS slowed it down. Similarly, anodal cerebellar tDCS has been shown to enhance reach adaptation in older adults and may compensate for the age-related impairment of motor learning [7]. Beneficiary cerebellar tDCS effects have also been described in the acquisition of conditioned eyeblinks [8]. Comparatively few studies investigated the effects of cerebellar tDCS on the acquisition of new motor skills. Anodal and cathodal cerebellar tDCS led to an improvement in a skilled ankle motor tracking task [9]. Anodal cerebellar tDCS improved also acquisition of a sequential visual isometric pinch task [10].

The cerebellum is part of the motor skill learning network also comprising the primary, premotor and supplementary motor areas (SMA), parietal cortex and the basal ganglia [11]. Imaging data show that the cerebellum is of particular importance in the early stages of learning [12, for review]. It may also contribute to cognitive strategies in early learning because of the known connections of the posterolateral cerebellar hemispheres to prefrontal areas, [13–14].

In the present study the impact of cerebellar tDCS on learning of a complex whole body motor skill was investigated in young and healthy subjects. This task requires highly coordinated whole-body movements. It was chosen in order to model complex motor behavior that is essential for posture and balance functions in everyday life [15–16]. A previous study has shown that concomitant anodal tDCS of SMA and cathodal tDCS of the prefrontal cortex impaired learning in this task in young and healthy subjects [16]. tDCS may be more beneficial applied to other brain regions likely involved in the task, in particular the cerebellum. If applicable, cerebellar tDCS could be a prospective tool in ameliorating postural instability in cerebellar patients based on the combination of rehabilitation training and stimulation.

## Materials and Methods

A total of 40 healthy subjects took part in the study. Five subjects had to be excluded because of technical problems. Another five subjects had to be excluded, because of study protocol violations. Data of 30 healthy subjects (15 male, 15 female, mean age 23.7 ± 2.4 years, range 20–30 years) were included in statistical analysis. Subjects with a history of neurologic, psychiatric or orthopedic diseases were excluded. In addition, subjects who practice or practiced board sports were excluded. None of the participants was taking centrally acting medications. All subjects underwent a full neurological examination before the start of the study. Neurological examination was unremarkable in all subjects. Written informed consent was taken from all subjects. The study was approved by the ethics committee of the medical faculty of the University Duisburg-Essen and conducted in accordance to the Declaration of Helsinki.

**Fig 1:**
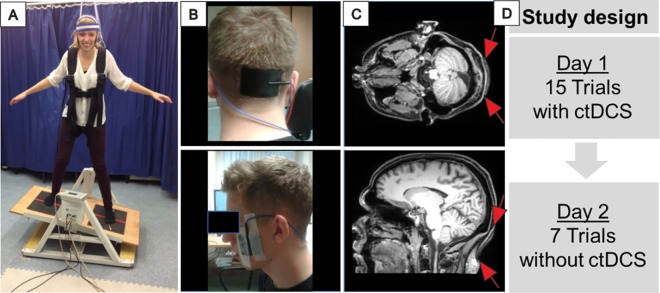
Experimental design. **A:** Subject performing the dynamic balance task on a Lafayette Instrument 16030 stability platform®. The individual shown in this figure has given written informed consent (as outlined in PLOS consent form) to publish these case details. Subjects were instructed to hold the platform in a horizontal position as long as possible, **B:** tDCS electrode position The top end of the cerebellar electrode (width 7 cm by height 5 cm) was centered 2 cm below the inion. Two reference electrodes were placed over the buccinator muscles bilaterally (5 cm x 5 cm). **C:** Position of the cerebellar electrode as revealed by axial and sagittal T1-weighted magnetic resonance imaging (MRI) scans in a healthy subject. Electrode paste leads to hyperintensive MRI signal. Position is indicated further by red arrows, **D**: Study design: Two days of training, cerebellar tDCS (ctDCS) was applied only on day 1. There were 15 trials on the first day and 7 trials on the second day of training, 30 seconds each.

Balance training was performed on a Lafayette Instrument 16030 stability platform® (**Fig 1A**) on two consecutive days. The platform was freely movable to the right and to the left. Foot position was fixed. Subjects were instructed to hold the platform in a horizontal position as long as possible. In order to avoid free fall, subjects wore a loosely fitted safety harness. Training consisted of 15 trials, 30 seconds each, on day 1 and 7 trials on day 2. Between trials there were rest periods of 10 seconds to avoid muscle fatigue with a longer interval of 20 seconds after the seventh trial on day 1. During rest periods the platform was lowered to the ground (side alternated between trials).

Participants received cerebellar tDCS during training on day 1 only. Duration of the stimulation was 10 minutes (15 trials, 30 seconds each, 10 seconds rest time between the trials with a longer rest of 20 seconds after the seventh trial). Effects of retention were tested on the second day. Cerebellar tDCS was applied using a Neuroconn® DC stimulator (current intensity 2.8 mA). Participants received either anodal, cathodal or sham stimulation (5 male and five female subjects in each subgroup). In sham stimulation current was ramped-up in 30 seconds, remained at 2 mA for a duration of 20 seconds, after which current was ramped down again for the remainder of the experiment. The study was double-blinded. Because the task involved learning of balance and motor strategies cerebellar tDCS included stimulation of the midline and posterolateral cerebellar hemispheres. The cerebellar electrode (width 7 cm by height 5 cm) was centered 2 cm below the inion (that is, the top end was 0.5 cm above the inion) [17]. Different to Ferrucci et al. [17] two reference electrodes were placed over the buccinator muscles bilaterally (5 x 5 cm^2^). Electrodes were fixed with Ten20® conductive paste (Neurodiagnostic Electrode Paste, Weaver and Company, USA) and tapes around the head. At the beginning of the experiment participants performed one test trial without cerebellar tDCS to exclude differences in baseline performance between the three stimulation groups.

Mean platform angle deviation and mean balance time were assessed in each trial as measures of balancing ability. Mean balance time was included to allow for direct comparison with previous studies using the same task [15–16]. The platform angle is expressed as voltage between 0 and 5 volts and recorded by an analog-to-digital converter (National Instruments, Germany) at 1 kHz. Horizontal position was controlled and zeroed by means of a spirit level. Balance time was defined as the time during one trial, in which subjects were able to hold the platform between -5° to 5° degrees relative to earth horizontal (0°), i.e. the amount of time during the trial that they were within this boundary. Thus, mean balance time is based on the dichotomization of a continuous measure (see [18], for discussion of limitations).

### Statistical analysis

Mean balance time and the mean platform deviation in the test trial without cerebellar tDCS were compared between stimulation groups using one-way analyses of variance (ANOVA). ANOVA with repeated measures were calculated with mean balance time and mean platform angle deviation as dependent variable, trial (1–15: day 1, 1–7: day 2) as within subject factor and stimulation group (anodal vs. cathodal vs. sham) as between subject factor. First, analyses were performed separately for the two days of training. Second, to assess effects of retention, day (day 1 vs. day 2) was considered as additional within subject factor comparing the last seven trials on day 1 and the seven trials on day 2. Differences were considered to be significant at p < 0.05. For all effects, the degrees of freedom were adjusted, if appropriate, according to Greenhouse and Geisser. ANOVAs were calculated using SPSS software (version 17, IBM Company, New York, USA).

Equivalence tests were performed on mean differences between sham and stimulation groups using the Two One-Sided Tests (TOST) procedure [19–20]. Equivalence was concluded if the 90% of the confidence intervals (CIs) for the differences between sham and stimulation groups means fell within the range of ± one standard deviation of the respective mean of the sham group at the end of the day 1 or day 2, respectively.

## Results

**Fig 2:**
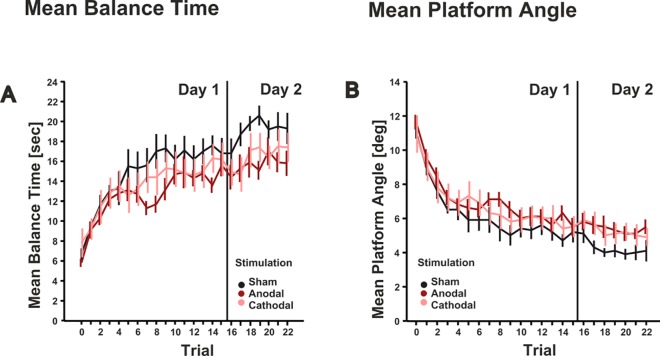
Mean balance time and standard error (**A**) and mean platform angle and standard error (**B**) are shown across trials on the two days of training (day 1: trial 1–15, day 2: trial 16–22) in the three stimulation groups (sham: black lines; anodal: dark red lines, cathodal: light red lines). Trial 0 indicates the test trial without cerebellar tDCS.

Performance in the test trial without cerebellar tDCS (trial 0 in **Fig 2**) was not different between the three stimulation groups [mean balance time: F(2,27) = 1.182, p = 0.322; mean platform angle: F(2,27) = 0.286, p = 0.753; one-way ANOVA].

All subjects showed a significant increase of balance time (**Fig 2A**) and a significant decrease of mean platform angles (**Fig 2B**) across trials on day 1, retained learned balance abilities on day 2 and showed further improvement on day 2 [trial effects, all p values < 0.001; ANOVA with repeated measures, for details see **Tables 1**and **2**].

**Table 1:** Summary of statistical findings considering mean balance time.

Mean Balance time	Stimulation effect	Trial effect	Stimulation by trial effect	Stimulation by day effect	Stimulation by day by trial effect
**Both genders**					
**Both days**	F(2,27) = 1.731	F(1,21) = 18.689	F(2,42) = 0.981	F(2,2) = 0.795	F(2,12) = 0.892
	p = 0.196	p < 0.001	p = 0.487	p = 0.462	p = 0.557
**Day 1**	F(2,27) = 1.139	F(1,14) = 15,374	F(2,28) = 1.065	n. a.	n. a.
	p = 0.335	p < 0.001	p = 0.379		
**Day 2**	F(2,27) = 2.935	F(1,6) = 3.489	F(2,12) = 0.579	n. a.	n. a.
	p = 0.07	p = 0.003	p = 0.857		
**Male subjects only**					
**Both days**	F(2,12) = 1.344	F(1,21) = 10.016	F(2,42) = 0.746	F(2,2) = 8.172	F(2,12) = 0.385
	p = 0.297	p < 0.001	p = 0.874	p = 0.014	p = 0.965
**Day 1**	F(2,12) = 0.995	F(1,14) = 7.551	F(2,28) = 0.834	n. a.	n. a.
	p = 0.398	p < 0.001	p = 0.706		
**Day 2**	F(2,12) = 1.802	F(1,6) = 2.8	F(2,12) = 0.684	n. a.	n. a.
	p = 0.207	p = 0.017	p = 0.761		
**Female subjects only**					
**Both days**	F(2,12) = 1.962	F(1,21) = 9.055	F(2,42) = 0.796	F(2,2) = 0.693	F(2,12) = 0.665
	p = 0.183	p < 0.001	p = 0.812	p = 0.519	p = 0.779
**Day 1**	F(2,12) = 1,521	F(1,14) = 8.106	F(2,28) = 0.913	n. a.	n. a.
	p = 0.258	p < 0.001	p = 0.595		
**Day 2**	F(2,12) = 2.538,	F(1,6) = 2.453	F(2,12) = 0.364	n. a.	n. a.
	p = 0.12	p = 0.033	p = 0.972		

**Table 2:** Summary of statistical findings considering mean platform angle.

Mean Platform angle	Stimulation effect	Trial effect	Stimulation by trial effect	Stimulation by day effect	Stimulation by day by trial effect
**Both genders**					
**Both days**	F(2,27) = 1.165	F(1,21) = 28.821	F(2,42) = 0.664	F(2,2) = 0.63	F(2,12) = 0.785
	p = 0.327	p < 0.001	p = 0.845	p = 0.54	p = 0.665
**Day 1**	F(2,27) = 0.783	F(1,14) = 24.376	F(2,28) = 0.76	n. a.	n. a.
	p = 0.467	p <0.001	p = 0.692		
**Day 2**	F(2,27) = 1.941	F(1,6) = 3.416	F(2,12) = 0.309	n. a.	n. a.
	p = 0.129	p = 0.003	p = 0.987		
**Male subjects only**					
**Both days**	F(2,12) = 1.243	F(1,21) = 16.124	F(2,42) = 0.929	F(2,2) = 0.249	F(2,12) = 0.387
	p = 0.323	p < 0.001	p = 0.6	p = 0.784	p = 0.964
**Day 1**	F(2,12) = 1.053,	F(1,14) = 12.814	F(2,28) = 1.164	n. a.	n. a.
	p = 0.379	p < 0.001	p = 0.273		
**Day 2**	F(2,12) = 1.528	F(1,6) = 3.308	F(2,12) = 0.567	n. a.	n. a.
	p = 0,256	p = 0.006	p = 0.862		
**Female subjects only**					
**Both days**	F(2,12) = 1.777	F(1,21) = 14.736	F(2,42) = 1.25	F(2,2) = 0.556	F(2,12) = 0.683
	p = 0.211	p < 0.001	p = 0.152	p = 0.031	p = 0.762
**Day 1**	F(2,12) = 1.651	F(1,14) = 13.561	F(2,28) = 1.473	n. a.	n. a.
	p = 0.233	p < 0.001	p = 0.071		
**Day 2**	F(2,12) = 1.941	F(1,6) = 2.047	F(2,12) = 0.48	n. a.	n. a.
	p = 0.186	p = 0.07	p = 0.92		

**Fig 3:**
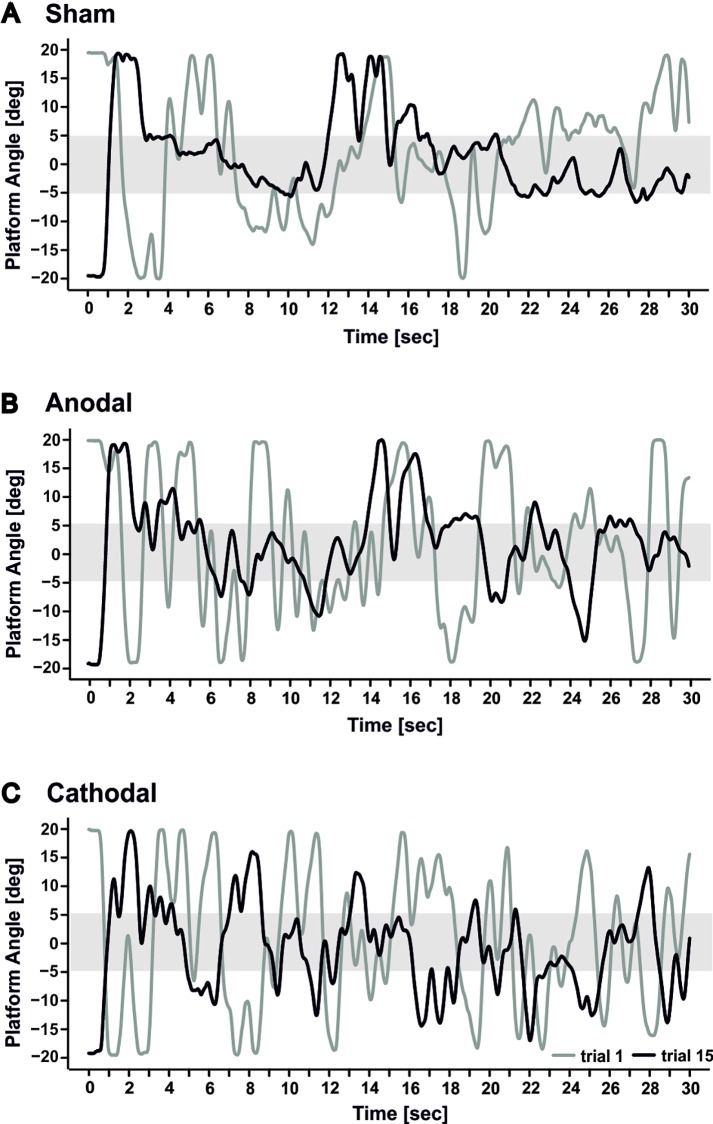
Individual platform angle traces are shown of characteristic subjects in each stimulation group (**A**: sham; **B**: anodal; **C**: cathodal) in the first trial (trial 1;grey lines) and the last trial (trial 15; black lines).

Learning rate was not different between the three modes of stimulation neither on day 1 nor on day 2 (trial by stimulation interaction effects, all p values > 0.19). Likewise, individual traces in three characteristic subjects show a marked reduction of platform angle comparing trial 15 and trial 1 on day 1 regardless of the mode of stimulation (**Fig 3**). It has to be noted, however, that mean differences between the sham and verum groups at the end of days 1 and 2 did not prove equivalence (p > 0.05) (see **Figure A and Table A** in S1 File).

Performance in the anodal and cathodal groups tended to be worse compared to sham on day 1, and, more pronounced, on day 2, both regarding mean balance time and mean platform angle. On day 1 stimulation effects were not significant [mean balance time: F(2,27) = 1.139, p = 0.335; mean platform angle: F(2,27) = 0.783, p = 0.467]. On day 2 the stimulation effect was close to significance regarding mean balance time [mean balance time: F(2,27) = 2.935, p = 0.07; mean platform angle: F(2,27) = 1.941, p = 0.129].

Statistical comparison of balance parameters on day 1 and day 2 (last seven trials on day 1 compared with the seven trials on day 2 showed significant trial effects (p values < 0.028), but no significant trial by day interaction effects (p values > 0.129). Although performance was on average lower in the anodal and cathodal groups compared to the sham group stimulation effects did not reach significance [mean balance time: F(2,27) = 1.821, p = 0.181; mean platform angle: F(2,27) = 1.418, p = 0.26]. Stimulation by day interaction effects, and stimulation by day by trial interaction effects were not significant (all p values > 0.114; for details see **Tables 1**and **2**).

**Fig 4:**
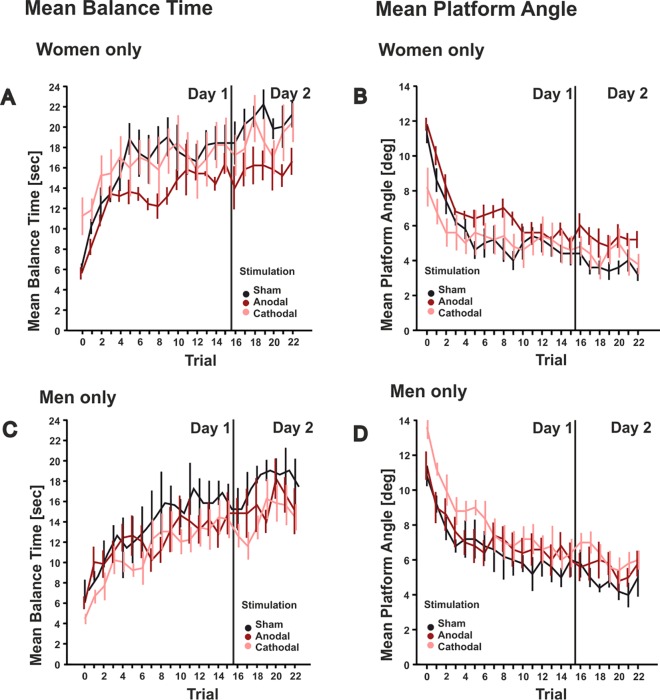
Mean balance time and standard error (**A**, **C**) and mean platform angle and standard error (**B**, **D**) are shown across trials on the two days of training (day 1: trial 1–15, day 2: trial 16–22) in the three stimulation groups (sham: black lines; anodal: dark red lines, cathodal: light red lines) separated by gender (**A**, **B**: women only; **C**, **D**: men only). Trial 0 indicates the test trial without cerebellar tDCS.

Finally, female and male participants were analyzed separately (**Fig 4**). As expected, trial effects were significant in female and male subjects considering both parameters and both days (all p values < 0.033). Stimulation by trial interaction effects were not significant neither in male nor female subjects (all p values > 0.12). Again, performance tended to be worse in the anodal and cathodal subgroups compared to sham stimulation on both days, but the difference did not reach significance neither in male nor in female subjects (all p values > 0.12).

Male subjects, however, performed generally worse than female subjects. Statistical comparison of all male and female subjects across trials on both days showed significant gender effects [mean balance time F(1,28) = 5.839, p = 0.022; mean platform angle: F(1,28) = 7.216, p = 0.012]. Male subjects [mean 181.3 standard deviation (SD) 8.8 cm, range 166–198 cm] were on average taller than female subjects (mean 170 SD 5.3 cm, range 160–180 cm; p < 0.001). Considering all male and female subjects, body height correlated negatively with mean balance time (r = -0.353, p < 0.001; bivariate Pearson’s correlation) and positively with mean platform angle (r = 0.354, p < 0.001). Thus, worse performance in male compared to in female subjects is likely explained by the combination of fixed foot position and higher center of mass in the taller male subjects.

## Discussion

Young healthy subjects showed significant effects of learning in a complex whole body dynamic balance task. They were able to increase the time they were able to stand on a freely moving platform and to decrease the mean platform angle. Motor performance improved on day 1, learned motor skill was retained overnight and subjects showed further improvements on day 2. No significant effects of cerebellar tDCS, however, were observed. In particular, learning rate did not increase during anodal tDCS and did not decrease during cathodal tDCS on day 1. Neither male nor female participants showed significant cerebellar tDCS effects. Possible reasons for the lack of cerebellar tDCS effects will be discussed below.

Firstly, cerebellar tDCS effects and tDCS effects in general are likely task dependent. The most prominent effects of cerebellar tDCS have been described in adaptation of reaching movements to visuomotor perturbations [5], locomotor adaptation [6] and in eyeblink conditioning [8]. Although the cerebellum likely contributes to motor skill acquisition [12; 21], at least in the early stages, the relative contribution of the cerebellum to motor learning may be most prominent for motor adaptation and associative learning. Therefore, adaptation and associative learning tasks may be more sensitive to cerebellar tDCS effects. Learning of a complex motor skill may be more sensitive to tDCS effects of other brain areas involved in motor learning. A recent study by Kaminski et al. [16], however, was unable to show beneficiary effects of tDCS of the SMA and prefrontal cortex (PFC) using the same complex whole body dynamic balance task as in the present study. Rather cathodal stimulation of the (right) PFC impaired motor learning. Likewise, subjects tended to perform worse who received verum stimulation compared to sham stimulation in the present study. This difference, however, was not significant. tDCS effects may have been more prominent in Kaminski et al. [16] because of small differences in age (subjects in the present study were on average 1.9 yrs. younger) and longer rest periods between trials (10 sec. in the present study compared to 90 sec. in [16]). Performance was generally better in the present group, and may have led to more pronounced ceiling effects (compare **Fig 2**of the present paper and Fig 2 in [16]). Overall, postural balance function might be less susceptible to changes of cerebellar and cerebral excitability than arm movement and locomotor tasks.

Secondly, cerebellar tDCS stimulation parameters may have been suboptimal for the given task. We used the same size and position for the active electrode as in Ferrucci et al. [17]. The current intensity and location of the return electrode, however, were different. Because of the dynamic balance task is a complex task, which involves posture and control of both sides of the body, our aim was to stimulate the cerebellar midline and both hemispheres. To emphasize stimulation of the cerebellar midline, two reference electrodes were used over the buccinators muscles. Cerebellar tDCS effects may be more prominent using unilateral stimulation [5–6;8;10]. Current intensity, density and the time of stimulation was comparable with previous studies. It cannot be excluded, however, that cerebellar tDCS effects would have been present using for example a longer time of stimulation or during repetitive stimulation sessions. Furthermore, the most critical cerebellar areas may have been at least partly spared. Strategic learning has been shown to be an important component to learn the present task [15]. Because of their known connections with association cortices the posterolateral cerebellar hemispheres are most likely to contribute to strategic learning. Although a previous modelling study that used the same size and position of the active electrode as in the present study, showed that the tDCS field distribution covered both the cerebellar midline and major parts of both cerebellar hemispheres, stimulation density of the most posterolateral parts of the hemisphere may have been insufficient (see Fig 2 in [17]). In the future, studies modelling current flow as well as using neurophysiological measures (including cerebellar brain inhibition, CBI) would be of interest to compare the effectiveness of different cerebellar tDCS montages used in the literature [5–6;22] with our montage.

Thirdly, as briefly mentioned above, ceiling effects may have been present in young and healthy subjects. Thus, in particular given the nature of the task (imminent loss of balance) subjects may have performed at maximum possible learning level. This may be different in elderly subjects or in subjects with neurological disease. Cerebellar volume and motor learning are known to decline with increasing age and in cerebellar degeneration [23–25].

Fourthly, based on structural brain imaging data by Taubert and coworkers [15] one may argue that acquisition of the whole body dynamic balance task does not dependent on the cerebellum. The authors reported that grey matter (GM) volume in the left cerebellum correlated negatively with improvements in motor performance using the same task as in the present study. Likewise, they found a GM decrease in lobule VIII in the cerebellum bilaterally, and a mean diffusivity (MD) increase in right cerebellar white matter (WM) regions. These findings, however, do not allow the conclusion that the cerebellum is not involved in this complex motor learning task. Firstly, this reduction did occur only in the later phases of a six weeks learning period. Similar to learning of other motor skills, the cerebellum may be more important during the initial stages of learning [11–12]. Furthermore, as Taubert and coworkers pointed out in their supplementary materials “alternatively, synaptic pruning, decreased synapse head-size due to long-term depression or proliferation of intracortical axons could be discussed as possible mechanisms underlying grey matter reduction.”[15].

Finally, and maybe most importantly, tDCS effects may have been missed because of the small group size. However, on the observed effect size we calculated an a-priori power analysis. To achieve a power of 0.5 a total sample size of 474 would be required, to achieve a power of 0.95 a total sample size of 1449. On the other hand, a larger sample size with a narrower confidence interval (CI) may have shown equivalence, that is statistical confirmation for a lack of difference between stimulation groups [19–20]. Results need to be confirmed in future studies using significantly larger subject populations.

Despite these limitations we believe that it is important to report the negative results. Because it is difficult to statistically confirm negative findings, they are frequently not published. A recent meta-analysis questioned the reliability of tDCS effects [22]. This meta-analysis has serious methodological flaws and has widely been criticized [26]. It will be of interest to investigate the reproducibility of cerebellar tDCS effects in the future. It has recently been recognized, that individual differences in sensitivity to non-invasive brain stimulation may play a role [27].

In conclusion, cerebellar tDCS did not facilitate learning of a complex whole body dynamic balance task in young and healthy subjects. There are different reasons to explain the negative findings. Cerebellar tDCS effects may be task dependent that is cerebellar tDCS effects may be more beneficial in motor adaptation and associative learning tasks than during skill acquisition. On the other hand, stimulation parameters, for example localization and size of the cerebellar tDCS electrode, may have been suboptimal for the given task. Furthermore, ceiling effects may be present in young and healthy subjects. Finally, group size may have been too small. It cannot be excluded that cerebellar tDCS improves motor skill acquisition in larger groups of young subjects, in elderly subjects or in patients with cerebellar disease.

## Supporting Information

S1 File Legend explaining parameters in submitted data spreadsheets (Platform Angle-Data Spreadsheet, Balance Time-Data Spreadsheet) and Equivalence Test. (DOCX)

S2 File Informed Consent of Individuum shown in Fig 1. (PDF)

S1 Table Balance Time-Data Spreadsheet. (XLS)

S2 Table Platform Angle-Data Spreadsheet. (XLS)
