# Supplementary material for: Cerebellar tDCS Does Not Improve Learning in a Complex Whole Body Dynamic Balance Task in Young Healthy Subjects
Source: PLoS One. 2016 Sep 26;11(9):e0163598. doi: 10.1371/journal.pone.0163598 (PMC5036893; doi:10.1371/journal.pone.0163598)
Supplement: S1 File — (DOCX) [file pone.0163598.s001.docx]

**Legend explaining parameters in submitted data spreadsheets (Platform Angle-Data Spreadsheet, Balance Time-Data Spreadsheet)**

**Subj** = subject code

**Age** = subject´s age in years

**Gender**  m = male, f = female

**Cond1** = mode of stimulation on day 1 (anodal, cathodal, sham)

**Cond2** = mode of stimulation on day 2.

We had expected that anodal stimulation on day 1 lead to improved learning. In that case, we would have been interested whether or not these effects could be reversed by cathodal stimulation on day 2, Config2. Cond2 refers to the mode of stimulation at the end of day 2. At the beginning of day 2 seven trials were performed without stimulation. These trials are reported in the manuscript. On day 2, an additional seven trials have been performed. During these trials, 50% of the subjects received cathodal tDCS, and 50% sham. Because no tDCS effects were observed in Cond1, data in Cond2 were not further analyzed.

**Config1, Config2** = The Neuroconn® DC stimulator allows to save four stimulation configurations chosen by the examiner. Configuration B: anodal or sham stimulation; Configuration C: cathodal or sham stimulation. Config1: stimulation configuration on day 1; Config2: stimulation configuration on day 2.

**Code1, Code2** = The Neuroconn® DC stimulator allows for blinding of the examiner using 5-digit codes. A series of codes are provided by the manufacturer which code for verum or sham stimulation. Verum stimulation depends on the configuration (see Config1, Config2). The code assigned to each subjects are given in the data spreadsheet. Codes were assigned at random by another group member at the start of the study. Code1 = code used on day 1; Code2 = code used on day 2.

**D1U** D1 = day 1, U = Test trial without cerebellar tDCS

**D1T1 and following**  D1 = day 1, T1 = trial number (T1-T15)

**D2T1 and following** D2 = day 2, T1 = trial number (T1-T7)

**D2kT1 and following** D2 = day2, kT1 = trial number (kT1-kT7) with stimulation on day2 (see explanation for Cond2)

**Yellow fields** = Subjects, which were excluded from data analysis. Subjects #1, 2, 30, 35, and 39 had to be excluded because of study protocol violations.

**Note that** in five subjects (#23, 28, 34, 37 and 38) balance time and platform angle were not recorded because of technical problems. Numbers for these five subjects are missing in the spreadsheet.

**Equivalence test**

Equivalence tests were performed on mean differences between sham and verum groups using the Two One-Sided Tests (TOST) procedure (Schuirmann, 1987; Rogers et al., 1993). Equivalence was concluded if 90% of the confidence intervals (CIs) for the differences between sham and verum group means fell within the range of ± standard deviation of the respective mean of the sham group at the end of day 1 and day 2, respectively.

**Table A**. Equivalence test results for the mean platform angle of sham versus stimulation groups.

| **Day** | **Group** | | | | | | **Equivalence criterion^*^** | **Equivalence testing** | | | | | |
| --- | --- | --- | --- | --- | --- | --- | --- | --- | --- | --- | --- | --- | --- |
|  | **Sham^‡^** | | **Anodal^‡^** | | **Cathodal^‡^** | |  | **Anodal group** | | | **Cathodal group** | | |
|  | **Mean** | **SD** | **Mean** | **SD** | **Mean** | **SD** |  | **90% CI** | | ***p*^†^** | **90% CI** | | ***p*^†^** |
|  |  |  |  |  |  |  |  | **LCL** | **UCL** |  | **LCL** | **UCL** |  |
| 1 | 5.14 | 1.71 | 5.58 | 1.65 | 5.49 | 2.02 | ±1.71 | -1.74 | 0.871 | 0.054 | -1.79 | 1.11 | 0.059 |
| 2 | 4.28 | 1.73 | 5.35 | 1.48 | 4.93 | 1.59 | ±1.73 | -2.32 | 0.177 | 0.186 | -1.94 | 0.64 | 0.081 |

*Note.* SD – standard deviation, CI – confidence interval, UCL – upper confidence limit, LCL – lower confidence limit.

**^‡^** n=10

^*^Equivalence criterion is set as ± SD of the respective sham group.

**^†^**The highest *p* value of the two one-sided tests has been reported, *p* ≤ 0.05 for equivalency per each one-sided test.

**Fig A**. The 90% and 95% confidence intervals (CIs) around differences between sham and stimulation group means at the end of **A**) day 1 and **B**) day 2. The tick marks indicate the endpoints of 90% and the small circles indicate the endpoints of the 95% CIs. Equivalence of the means was established if the 90% CIs of the mean difference was within the acceptable difference interval defined as ± SD of the respective sham group mean (±1.71 and ±1.73 for Day1 and Day2, respectively). The groups do not prove equivalence (p>0.05), however, a larger sample with a narrower CI may have shown equivalence.
